# Tracking SARS-COV-2 variants using Nanopore sequencing in Ukraine in 2021

**DOI:** 10.1038/s41598-022-19414-y

**Published:** 2022-09-21

**Authors:** Anna Yakovleva, Ganna Kovalenko, Matthew Redlinger, Mariia G. Liulchuk, Eric Bortz, Viktoria I. Zadorozhna, Alla M. Scherbinska, Joel O. Wertheim, Ian Goodfellow, Luke Meredith, Tetyana I. Vasylyeva

**Affiliations:** 1 Medical Sciences Division, University of Oxford, Oxford, UK; 2 Division of Infectious Diseases and Global Public Health, University of California San Diego, San Diego, CA USA; 3 Division of Virology, Department of Pathology, University of Cambridge, Cambridge, UK; 4 Department of Biological Sciences, University of Alaska Anchorage, Anchorage, AK USA; 5 State Institution “L.V. Hromashevskyi Institute of Epidemiology and Infectious Diseases of the National Academy of Medical Sciences of Ukraine”, Kyiv, Ukraine

**Keywords:** SARS-CoV-2, Phylogenomics, Sequencing

## Abstract

The use of real-time genomic epidemiology has enabled the tracking of the global spread of severe acute respiratory syndrome coronavirus 2 (SARS-CoV-2), informing evidence-based public health decision making. Ukraine has experienced four waves of the Coronavirus Disease 2019 (COVID-19) between spring 2020 and spring 2022. However, insufficient capacity for local genetic sequencing limited the potential application of SARS-CoV-2 genomic surveillance for public health response in the country. Herein, we report local sequencing of 103 SARS-CoV-2 genomes from patient samples collected in Kyiv in July-December 2021 using Oxford Nanopore technology. Together with other published Ukrainian SARS-CoV-2 genomes, our data suggest that the third wave of the epidemic in Ukraine (June-December 2021) was dominated by the Delta Variant of Concern (VOC). Our phylogeographic analysis revealed that in summer 2021 Delta VOC was introduced into Ukraine from multiple locations worldwide, with most introductions coming from Central and Eastern European countries. The wide geographic range of Delta introductions coincides with increased volume of travel to Ukraine particularly from locations outside of Europe in summer 2021. This study highlights the need to urgently integrate affordable and easily scaled pathogen sequencing technologies in locations with less developed genomic infrastructure, in order to support local public health decision making.

## Introduction

The novel severe acute respiratory syndrome coronavirus 2 (SARS-CoV-2) was first identified in a cluster of pneumonia cases with an unknown cause in Wuhan, China in December 2019^1,2^. The virus quickly spread and reached pandemic status by March 2020; with over 519 million cases reported and resulting in over 6.26 million deaths globally as of May 11th 2022^3^. In just under a year since the beginning of the pandemic, several SARS-CoV-2 vaccines have been developed and deployed^4^. As of May 11th 2022, vaccination efforts have reached an estimated 67.2% of the global population, who have received at least one vaccine dose; however, only 17% of vaccine recipients so far reside in low-income countries^4,5^.

Genomic epidemiology has become a state-of-the-art approach for Coronavirus Disease 2019 (COVID-19) outbreak investigations, providing an evidence-based foundation for public health contingency measures such as travel restrictions and national lockdown^6,7^. Rapid SARS-CoV-2 genomic sequencing has been instrumental in informing public health systems of novel virus importations, informed the detection of transmission chains for contact tracing, and enabled rapid identification of novel variants of concern (VOC) that display altered transmission, immune evasion, and/or epidemiological properties^7–9^.

Ukraine, with a population of 42 million people, has reported 4.8 million cases of COVID-19 and over 105,000 deaths from March 2020 through February 2022^10^. On February 24th 2022 Ukraine has been invaded by Russian forces; the war disrupted health care systems and official COVID-19 statistics after this date became unavailable^11,12^. As of February 2022, Ukraine had one of the lowest rates of vaccination among middle-income countries with only 35.7% of the total population having received at least one dose of vaccine^13^. The national COVID-19 response in Ukraine has been complicated by the transition from a fully governmentally-controlled to an insurance-based healthcare system^14^; it was expected that health and economic consequences due to the COVID-19 pandemic would be severe in Ukraine^15^. The war will likely exacerbate the effects of the pandemic by disrupting healthcare in Ukraine, and displacing the population^11^.

In summer 2021, Ukraine was one of the few European countries to open its borders to international travelers, requiring a negative SARS-CoV-2 test pre-departure and purchase of private medical insurance for entry for foreign citizens^16^. With the aim of encouraging international tourism and supporting rapid economic recovery, businesses such as restaurants, bars, and nightclubs were permitted to operate at full capacity^17^. Over 4.2 million international tourists travelled to Ukraine in 2021 compared to 3.4 million in 2020, with higher proportion of foreign citizens arriving from Asian, African, and North American countries^18^.

Ukraine has a recent history of outbreaks of infections such as polio and measles,^19,20^, drug-resistant tuberculosis^21,22^, and numerous veterinary pathogens^23^, which are sustainably controlled in other countries. Furthermore, despite a high HIV and hepatitis C virus prevalence, infectious disease genomic epidemiology is currently not integrated into public health decision making in Ukraine^24,25^. Unlike other European countries, this is due to the unavailability of large-scale genetic sequencing capacity in Ukraine. As a result, over the course of the SARS-CoV-2 pandemic thus far, sequencing activities were only minimally supported by the government, and SARS-CoV-2 genomic epidemiology has not contributed to the public health response in Ukraine.

To date, only one study on the phylodynamic analysis of the SARS-CoV-2 sequences obtained from Ukrainian patients has been reported, describing the first four months of the pandemic. It showed that the initial rise in the number of COVID-19 cases was likely led by multiple independent introductions of the virus into Ukraine prior to border closures^26^, similar to the findings of the importation and circulation of SARS-CoV-2 lineages in the UK^7^. For the first time, our study aimed to build local and sustainable SARS-CoV-2 sequencing capacity and genomic epidemiology expertise in Kyiv, Ukraine using the Oxford Nanopore Technology (ONT) MinION sequencing platform. We generated and analysed the most recent SARS-CoV-2 genetic sequences obtained in Ukraine, and described patterns of Delta VOC introductions in summer 2021, integrating our findings with global surveillance data, thus providing an evidence-based framework for future public health decisions in Ukraine.

## Results

### Epidemiology of COVID-19 in Ukraine between March 2020 and February 2022

**Figure 1:**
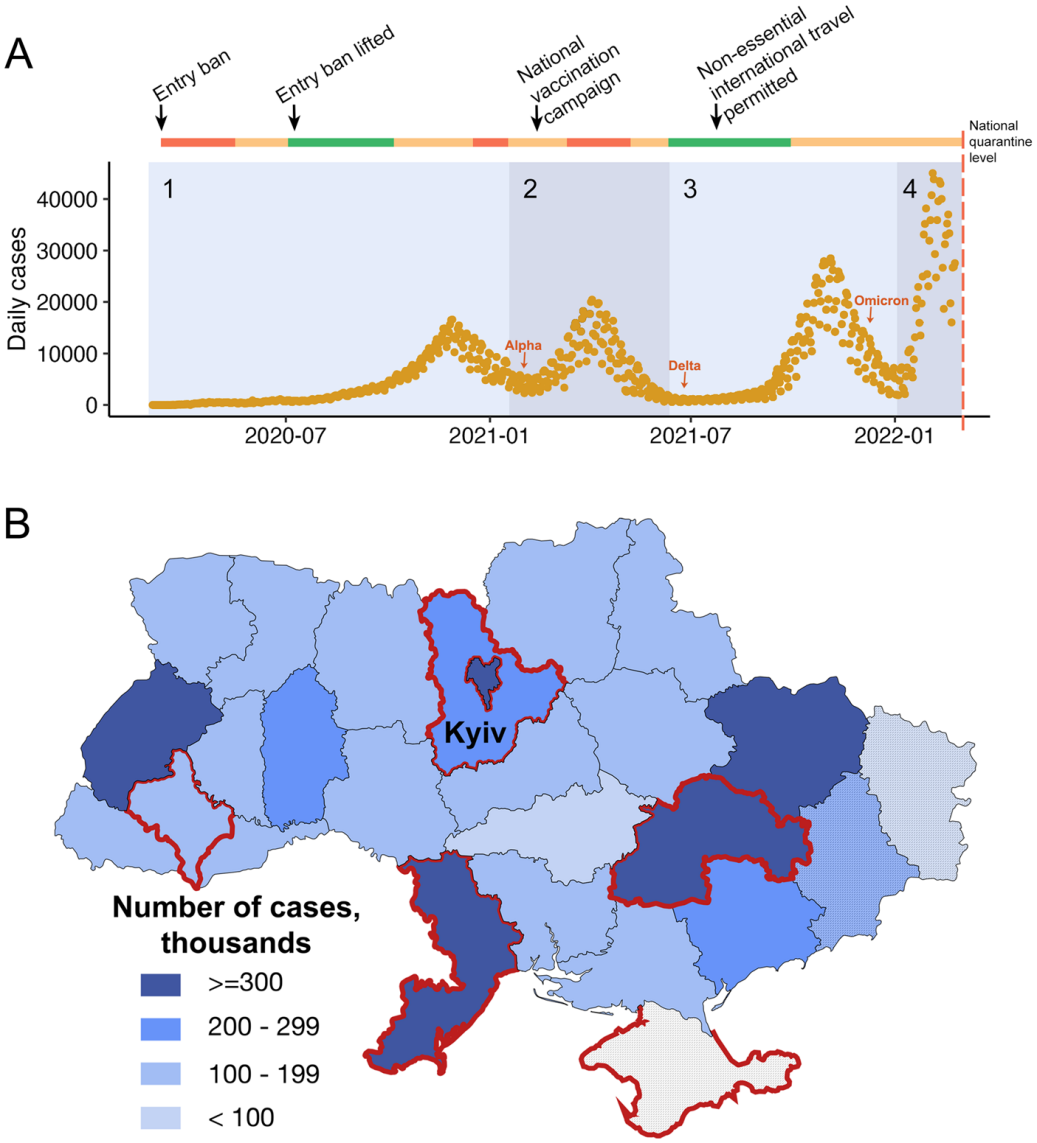
(**A**) Confirmed COVID-19 cases (orange) reported in Ukraine between March 2020 and February 2022 by The Ministry of Healthcare of Ukraine. (Four broad timeframes were assigned to indicate: “Pre-Wave” (March to 31 Aug 2020) marked together with “Wave 1" (01 Sep 2020–31 Jan 2021), “Wave 2” (01 Feb 2021–09 Jun 2021), “Wave 3" (10 Jun 2021 to 31 Dec 2021) and “Wave 4” (01 Jan 2022–22 Feb 2022). National quarantine measures, and implementation and easing of travel restrictions, are indicated along the colored bar on the top. The colors indicate national quarantine level at the time, with red representing complete national quarantine, orange representing adaptive quarantine with varied levels of restrictions in different regions, and green representing no or few restrictions. Detection of the first Alpha, Delta and Omicron variants in sequenced samples obtained from GISAID are indicated with red arrows. The red dashed line represents the beginning of the full scale Russian invasion of Ukraine, from which point no official statistics have been available. The graph was plotted in R Studio using the package ggplot2. (**B**) The distribution of COVID-19 cases by administrative regions in Ukraine. Regions not fully controlled by the Ukrainian government are marked with a striped pattern. Regions with > 40 sequences are highlighted in red.

According to the national COVID-19 tracking portal, Ukraine has experienced four waves of the epidemic: the first with a peak of 22,218 daily cases on November 26th, 2020, the second with a peak of 20,341 cases on April 4th, 2021, the third one with a peak of 27,377 on November 3rd, 2021, and the fourth one which peaked at 43,778 on February 3rd, 2022 (Fig. 1A)^27^. The distribution of cases by region was uneven, with the capital city of Kyiv, and major urban centers in Odesa, Kharkiv, Dnipro, and Lviv regions being most affected, with over 300,000 cases reported in each (Fig. 1B). Data from Donetsk and Lugansk regions are likely to be underestimated for the whole pandemic period due to incomplete data availability across these regions, whilst no data was available from Crimea, as reported by Ukrainian national public health organisations; no data was reported from Ukraine after February 22nd, 2022.

### SARS-CoV-2 genomic epidemiology in Ukraine

#### SARS-CoV-2 sequencing with MinION

**Figure 2:**
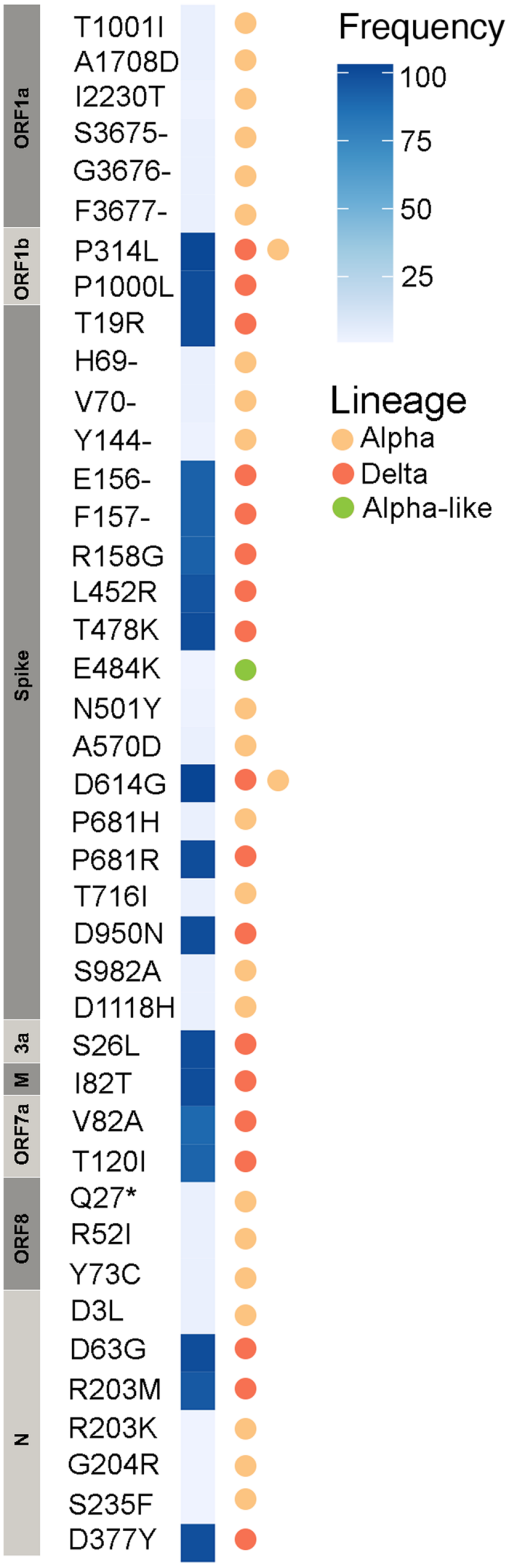
Heat map of frequency of amino acid mutations found in 103 SARS-CoV-2 genomes sequenced as part of this study from patients in Kyiv, Ukraine, in July and December 2021. Mutations associated with variant of concern lineages Alpha and Delta are indicated.

Using portable ONT MinION device and SARS-CoV-2 ARTIC Network workflow, we locally sequenced and analysed a set of 166 clinical samples (nasopharyngeal swabs) obtained from individuals who tested positive for SARS-CoV-2 by qRT-PCR in July (N = 24) and in October-December (N = 142) 2021 in Kyiv, Ukraine. All samples obtained in July resulted in high coverage (≥ 90% relative to the reference Wuhan-Hu-1 strain, N = 24) SARS-CoV-2 genomes. Of the 142 samples from October-December, 79 resulted in high coverage (≥ 90%) genomes and 63 produced partial genomes (37 samples resulting in 50–90% coverage and 26 samples < 50% coverage). In total, 103 high-coverage SARS-CoV-2 genomes from the third epidemic wave were obtained and further analysed in this study. Of those, 58% were obtained from female patients (see Supplementary Table S1 for additional information on the sequences and patients from whom they were sampled). We identified two variants of concern (VOC) in our samples: the Delta VOC (B.1.617.2-like) (N = 100) and the Alpha VOC (B.1.1.7-like) (N = 3) variants; all Alpha sequences were obtained from the samples collected in July. Virus genome analysis identified all common amino acid (aa) mutations which are associated with the Alpha and Delta VOC (Supplementary Table S1 and Fig. 2). Additionally, one sample assigned as an Alpha variant had the E484K mutation (G23012A) in the receptor binding domain (RBD) of the spike (S) protein and the N501S mutation instead of the N501Y mutation commonly found in this position for Alpha variant (Fig. 2). More details on the sequence quality and identified mutations can be found in the Supplementary Text.

#### SARS-CoV-2 lineages circulating in Ukraine

Next, we analysed SARS-CoV-2 lineage distribution using all available sequences (N = 883) obtained from patient samples collected in Ukraine in the GISAID database as of May 11th, 2022. Of those, 873 sequences collected between March 2020 and April 2022 were selected for further analysis of the SARS-CoV-2 lineage distribution in Ukraine (Supplementary Table S2); ten sequences were excluded due to an incomplete genome or lack of collection date information. The majority of selected sequences were sequenced locally in Ukraine (N = 520; 60%) using different sequencing platforms such as Ion Torrent, ONT MinION, and Illumina (MiSeq and iSeq100). Most (66%, N = 342) of the locally generated sequences were produced on the Ion Torrent platform, 14% (N = 73) were sequenced with the Illumina, and 20% (N = 105) with ONT MinION, of which 103 (98%) were sequenced in this study.

The SARS-CoV-2 lineage distribution in Ukraine consisted of five predominant lineages as designated by the PANGO nomenclature ^28^ (Supplementary Fig. S1): B.1 and its sub-lineages (N = 74), B.1.1 and its sub-lineages (N = 110), Alpha variant (N = 118) (including the B.1.1.7-like sequence with the E484K mutation), Delta variant (N = 466) and Omicron and Omicron-related lineages (N = 105) (Supplementary Fig. S1 and Supplementary Table S3). The 466 Delta sequences were assigned to 25 Delta VOC sub-lineages and 105 Omicron sequences were assigned to 13 Omicron VOC sub-lineages as described in Supplementary Table S3.

**Figure 3:**
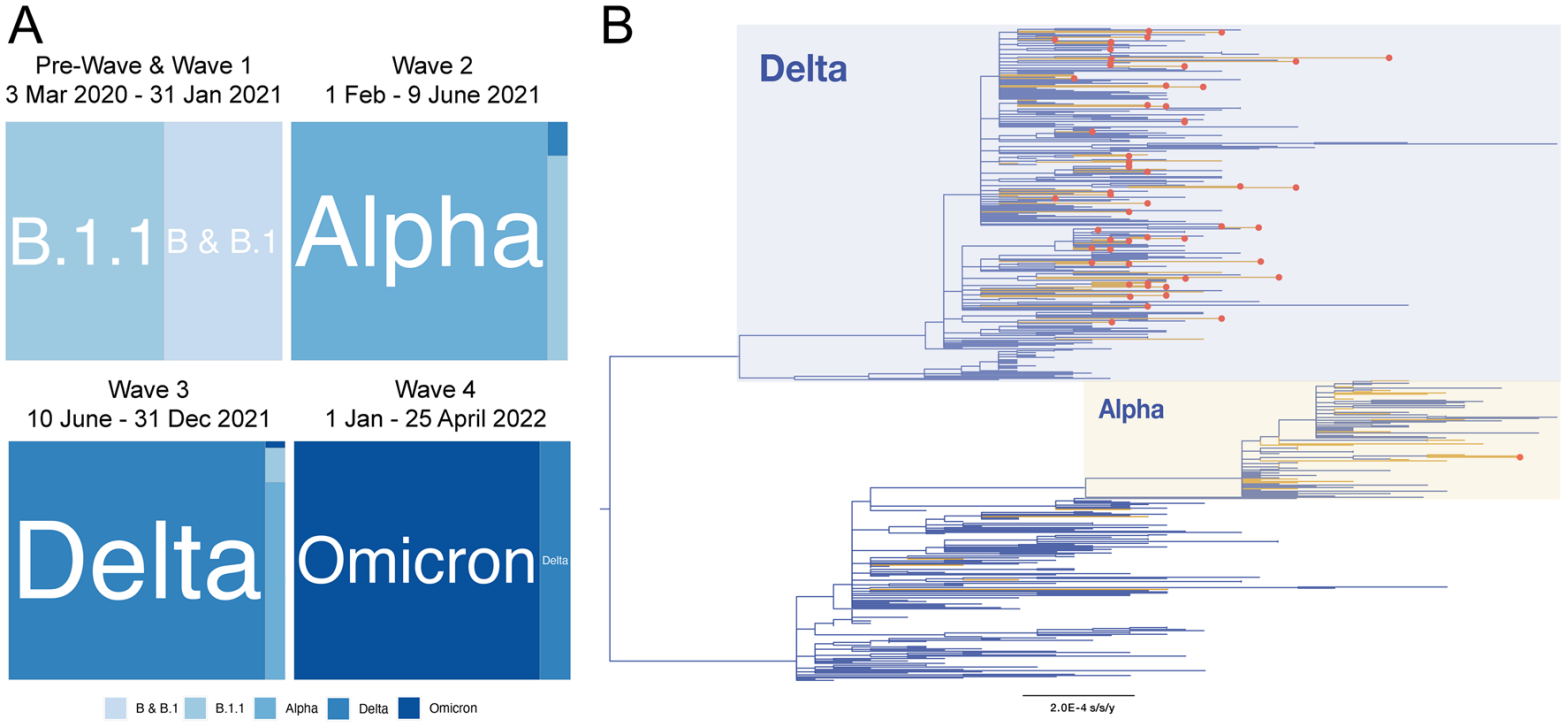
Phylogenetic analysis of the total SARS-CoV-2 lineages available from Ukraine. (**A**) Relative distribution of dominant SARS-CoV-2 PANGO lineages in epidemic waves of COVID-19 in Ukraine. “Pre-Wave” and “Wave 1” are combined into one square; (**B**) Maximum likelihood phylogenetic tree representing SARS-CoV-2 genetic diversity in Ukraine. Yellow branches correspond to sequences sampled in Kyiv. Red circles indicate genomes generated in this study by Nanopore sequencing. The bar at the bottom indicates genetic distance in substitutions per site per year. Branch tip labels are not shown for clarity of tree structure.

Lineage prevalence varied through the epidemic in the country, consisting of five broad time frames: a “Pre-Wave” (March to 31 Aug 2020) and “Wave 1" (01 Sep 2020–31 Jan 2021) dominated by B, B.1 and B.1.1, “Wave 2” (01 Feb 2021–09 Jun 2021) dominated by Alpha VOC, “Wave 3" (10 Jun 2021 to 31 Dec 2021) dominated by Delta VOC, and “Wave 4” (01 Jan–25 Apr 22 Feb 2022) dominated by Omicron (Fig. 3A). Despite the low number of sequences in comparison to confirmed infections (sequence data represent approximately 0.02% of COVID-19 cases), we found a diversity of B.1 and B.1.1, Delta and Omicron VOC sub-lineages in Ukraine (Supplementary Figs. S1 and S2; Supplementary Table S3). Among Delta VOC, the AY.122 sub-lineage dominant in Europe was most frequently detected (273 of 466 sequences, 58%) mainly in Wave 3, followed by B.1.617.2 originally identified in India (40 sequences, 8.5%), while 23 other Delta sub-lineages were found at 1–6% incidence. Omicron BA.1-related sub-lineage was first identified on December 19th, 2021 and then predominated in the first part of Wave 4 (January to February, 2022; 58 of 105 sequences, 55%) while the first confirmed BA.2 sub-lineage sequence was sampled on January 19th, 2022. The closest country of origin for all identified lineages is indicated in the Supplementary Table S3. Only 435 out of 873 (50%) Ukrainian sequences on GISAID were high-quality genomes as defined by GISAID and thus were included in the phylogenetic reconstruction of the SARS-CoV-2 genetic diversity in Ukraine, together with the 103 sequences generated in this study (N = 538, Fig. 3B).

### Phylogeographic analysis of the Delta variant sequences

To understand the timing and origin of Delta variant introductions into Ukraine in summer 2021, we performed a phylogeographic analysis of 1,733 Delta variant sequences. This included 56 Ukrainian sequences sampled in June–September 2021: 21 Delta sequences generated in this study and sampled in July 2021, 11 sequences from Crimea, and 24 other Delta sequences from Ukraine; and 1,677 sequences from other countries. Our analysis indicated at least 34 separate introductions into the country in summer 2021; most of the Ukrainian sequences were singletons on the global Delta diversity tree, but we also found 6 pairs, two clusters of five sequences, one cluster of three sequences, and one cluster of seven sequences (Supplementary Fig. S3). The phylogeographic analysis indicated the highest number of introductions from Turkey (four individual introductions) and at least one importation event from each of the Baltic countries, Slovenia, Nigeria, Jordan, Russia, Finland, Czech Republic, and United Arab Emirates. The TreeTime mugration analysis was not able to resolve the ancestral location for ten separate introduction events.

## Discussion

Ukraine has experienced four COVID-19 epidemic waves between the identification of the first case on February 29th 2020 and April 2022. Compared to other European countries, the first three epidemic waves in Ukraine lagged by several months as Ukraine did not experience many cases in spring 2020, likely due to a combination of a timely lockdown and a restriction of movement policy^29,30^. The border closure was applied to both foreign citizens and Ukrainian nationals in March 2020, three weeks after the first case was diagnosed in the country. However, lower numbers of registered cases in Ukraine may also be attributed to many infections likely being undiagnosed due to limited testing and diagnostic capacity in spring 2020.

When most of the world was approaching the second wave of the epidemic in September 2020, the number of COVID-19 cases in Ukraine started to grow quickly, resulting in the first epidemic wave in the country. Just as the epidemic waves in Ukraine followed a similar trajectory as in Western European countries, but with a few months delay, they have also been dominated by the same VOCs. As such, the second wave of the epidemic in Ukraine, which started in February 2021, was dominated by the Alpha VOC, and the third wave, which started in Ukraine in June 2021, by the Delta VOC, which remained dominant amongst the European Economic Area countries until September 2021^31^ and in Ukraine until December 2021. The fourth COVID-19 wave in Ukraine has been driven by Omicron BA.1 infections that likely peaked in February 2022 and were followed by an Omicron BA.2 wavelet. However, the true dynamics of the Omicron wave sub-lineages is impossible to determine due to the paucity of published genomics data from Ukraine since the Russian invasion on February 24th, 2022, with concomitant disruption of healthcare including disease surveillance, and large numbers of internally displaced people.

Unlike the strict measures in the beginning of the epidemic, no travel restrictions were imposed in summer 2021, and increased tourist flow likely contributed to the third wave of the epidemic in June-December 2021. Our phylogeographic analysis, which focused on Delta sequences available in Ukraine between June and September 2021, showed that the Delta variant has been introduced into Ukraine from multiple geographic locations. While this analysis suggested Delta VOC sub-lineages were introduced from nearby countries, such as Turkey, Russia, and Eastern European nations, we found evidence for introduction of sub-lineages from more distant locations in Africa and the Middle East. Interestingly, in summer 2021,  among the top-10 Delta case countries, introductions came only from two, from Turkey and US (see Methods). Such vast geographic spread of virus introductions into the country is likely the result of the increase in the tourist flow and the diversity of countries of origin of tourists travelling to Ukraine, as well as absence of COVID-19 restrictions in the country in summer 2021. Importantly, although we tried to find a balanced approach to sub-sampling of the global Delta variant diversity for our phylogeographic analysis, such inference is complicated by uneven testing and sequencing efforts across the globe, and by the fact that identical sequences can be found in the many countries considered in the analysis. Thus, results of our phylogeographic analysis should be interpreted with caution and only refer to the patterns observed in summer 2021 in the beginning of the third wave of the epidemic in Ukraine.

Large-scale genomic sequencing of SARS-CoV-2 has enabled global surveillance of pandemic dynamics but was yet to become part of the public health response in Ukraine at the time that it was invaded by Russia. In 2021, for the first time, we utilised ONT MinION technology in combination with ARTIC Network protocols to locally sequence 103 SARS-CoV-2 genomes collected from patient samples in Kyiv, Ukraine, to enable development of genomic epidemiology expertise and sequencing capacity in Ukraine. While most of the genomes sequenced with MinION were complete and of high-quality, some of the sequences produced in this study had lower than optimal genome coverage (< 90%), likely due to poor sample quality or low amplification efficiency where the amplicons are not equally amplified across all regions of the genome. The ARTIC V3 primer set was used for the 24 samples collected in July 2021, which has been previously reported to be associated with the amplicon 72 (S gene) dropout in the Delta variant sequences as observed here. The second major amplicon dropout reported for these sequences (amplicon 64 in ORF1ab) was likely due to a similar issue in primer compatibility. This prompted the development of the ARTIC V4 primer set compatible with the novel mutations^32^. Thus, ARTIC V4 primers were used to sequence samples collected in October–December 2021, which improved the coverage of Delta VOC and helped to significantly reduce the amplicon 64 and 72 dropouts.

Genomic epidemiology integration within public health response allows tracking of variants and mutations that potentially affect vaccine efficacy. For example, the E484K mutation in the S protein, an escape mutation that shows evidence of impacting the immune response and likely reducing vaccine effectiveness^33^, was identified in one Alpha variant sequence in our analysis. This mutation is rare for this lineage and is usually present in other VOC (Beta, Gamma, Zeta, and Eta)^34^. As of May 11th, 2021, the E484K mutation was identified in 3,786 (0.32%) of all Alpha sequences in GISAID, of which 0.23% were from Europe predominantly in France, Germany, Sweden and Denmark. The N501S mutation in S, another mutation found in one of our Alpha variant sequences, was also reported to increase receptor-binding domain (RBD) stability and RBD-ACE2 (angiotensin converting enzyme 2) binding affinity compared to the original Wuhan-Hu-1 isolate but was predicted to have a neutral effect on protein^35^. Furthermore, the same sample sequence had the Y144- deletion in the S protein associated with antibody escape^36^. Tracking of the prevalence of these mutations in Ukrainian SARS-CoV-2 viral population is of immense importance for the country’s vaccine strategy going forward, but is likely to be of low priority at the time that the country is experiencing an on-going war and humanitarian crisis^11^.

Vaccination in Ukraine against COVID-19 is among the lowest in Europe, with 15.2 million people fully vaccinated (34.3% of the population), 35.7% partially vaccinated, and 1.7% given a booster dose as of February 27th, 2021^3^. Without domestic vaccine manufacturing, Ukraine has relied on donations and external sourcing, for example, the WHO COVAX program that supplied 4.2 million doses total of AstraZeneca AZD1222 ChAdOx1, Pfizer-BioNTech BNT162b2 mRNA, and Moderna mRNA-1273 vaccines^37,38^. Vaccination began in February 2021, with half of the vaccine doses having been adminisered primarily to adults aged 20–49, and the remainder to those over 50 + years, with a small proportion of teenagers.

The Delta variant, which dominated the third wave of the epidemic in Ukraine, is characterized by increased transmissibility and virulence, and marginally lower vaccine efficacy (VE) of multiple vaccines against symptomatic COVID-19^4,39^. In combination with waning immunity against SARS-CoV-2^4,40,41^, the WHO recommended booster vaccination to enhance protection against the Delta VOC^37^. Despite the evidence that Delta variant was the dominant lineage in the third COVID-19 wave in Ukraine (Fig. 3A), only a negligible number of people in Ukraine (9,515, 0.02% of the population) received a booster vaccine dose as of December 31st, 2021, the end of the third wave of the epidemic in Ukraine. This was likely due to the limited availability of VOC surveillance to provide evidence of the virus strains circulating patterns, as well as access to more vaccine doses in this lower-middle income country.

While it is unlikely that genomic surveillance can be scaled up in Ukraine at the time of a crisis such as the ongoing war, our findings suggest that there is a pressing need in lower- and middle-income countries for input from genomic epidemiology to inform public health policy of the threat of emerging variants. In Ukraine, availability of national genomic surveillance earlier in the COVID-19 epidemic may have influenced better interventions, such as alternative timing of lifting of local restrictions and better regulation of international travel, thus slowing the spread of the VOCs in Ukraine. After the war, parallel to other efforts in restoration of health care systems in Ukraine, future investment in establishing a sustainable and local genomic surveillance program for SARS-CoV-2 and other prevalent pathogens will ensure evidence-based public health decision-making in Ukraine.

## Methods

### Sample collection

The State Institution “L.V. Hromashevskyi Institute of Epidemiology and Infectious Diseases of the National Academy of Medical Sciences of Ukraine” in Kyiv, Ukraine, obtained a randomised collection of COVID-19 patient nasopharyngeal swab samples from a private laboratory biobank and from the State Institution "Kyiv City Center for Control and Prevention of Diseases of Ministry of Health of Ukraine". Samples were collected in Kyiv between July 20–25, 2021 (N = 24) and in October-December 2021 (N = 142). The infection was confirmed by qRT-PCR using Abbott real-time SARS-CoV-2 amplification kit at the Hromashevskyi Institute. All study protocols were performed in accordance with the relevant guidelines and regulations and were approved by The Committee on Medical Ethics and Deontology of the State Institution " L. Hromashevskyi Institute of Epidemiology and Infectious Diseases of the National Academy of Medical Sciences of Ukraine", Kyiv, Ukraine (reference number: No.5 dated 07 October 2021). The requirement for written informed consent was waived by The Committee on Medical Ethics and Deontology of the State Enterprise “Institute of Epidemiology and Infectious Diseases named after L.V. Hromashevskyi of the National Academy of Medical Sciences of Ukraine”, Kyiv, Ukraine (reference number: No.5 dated 07 October 2021) since only anonymized and deidentified samples provided for clinical testing were used for genetic sequencing.

### Oxford Nanopore SARS-CoV-2 sequencing

Sequencing was performed at the Hromashevskyi Institute of Epidemiology and Infectious Diseases in August 2021 (N = 24; for the samples collected in July 2021) and in December 2021 (N = 142; for the samples collected in October-December 2021). Viral RNA was extracted from 140 μL patient sample using the QIAamp Viral RNA Mini kit according to the manufacturer’s instructions (Qiagen). In August, samples were sequenced using the NEBNext ARTIC SARS-COV-2 Companion Kit for Oxford Nanopore (New England BioLabs) with ARTIC nCoV-2019 v3 primers (https://www.protocols.io/view/ncov-2019-sequencing-protocol-v3-locost-bh42j8ye) as per the manufacturer’s instructions. In December, ARTIC nCoV-2019 v4 primers (https://github.com/artic-network/primer-schemes) were used for accurate sequencing of the Delta VOC spike region. Briefly, cDNA was generated using random hexamers, and the full-length genome was amplified with a multiplex PCR approach consisting of 400 bp overlapping amplicons. Subsequently, samples were purified with SPRI beads and uniquely labelled using the Native Barcoding Expansion kits Oxford Nanopore EXP-NDB104 (1–12) and EXP-NBD114 (1–24) in August and EXP-NBD196 (1–96) in December. Finally, libraries were prepared, each containing 24 or 96 samples, by ligation of Oxford Nanopore sequencing adapters and quantified using Quantus Flurometer (Promega). Libraries were sequenced using new MinION flow cells version 9.4.1 (ONT).

### SARS-CoV-2 genome assembly

Genomes were assembled using the ARTIC bioinformatics pipeline with a 20X minimum coverage across all genomic regions. First, basecalling was performed with guppy 5.0.11 using the dna_r9.4.1_450bps_sup.cfg model followed by demultiplexing with guppy 5.0.11 for barcode kits EXP-NBD104, EXP-NBD114 and EXP-NBD196. Then read filtering was performed with Artic Guppyplex allowing for read length between 400 and 700 nucleotides. Finally, ARTIC Minion nanopolish pipeline was run with the option to normalise to 200. All sequences obtained are available on GISAID EpiCov database (www.gisaid.org). The accession numbers of the sequences obtained in this study are presented in Supplementary Table S1.

### Phylogenetic analysis

We downloaded all complete SARS-CoV-2 genomes from Ukraine from GISAID database on May 11th, 2022. All Ukrainian sequences, from GISAID and generated in this study, were aligned using MAFFT. We masked all problematic sites in the alignment as suggested by De Maio et al. (https://virological.org/t/masking-strategies-for-sars-cov-2-alignments/480). PANGO lineage assignments were determined using the Pango v.4.0.6 nomenclature tool^28^. Mutation calling and sequence quality were analysed using Nextclade (https://clades.nextstrain.org/) and Geneious Prime 2021.2.2 software (Biomatters, New Zealand). Relative distribution of dominant lineages and mutation heatmap was made using R Studio with the ggplot2 package. A maximum likelihood phylogenetic tree was then built using only high-quality genomes with IQTREE2 program under the general time reversible model allowing for rate heterogeneity among sites and proportion of invariable sites (GTR + G + I).

### Phylogeographic analysis

To establish the number and the source country of the Delta variant introductions into Ukraine in summer 2021, we have downloaded all complete high-quality with available sampling date Delta variant sequences sampled between January 1st and September 30th available on GISAID on September 30th (N = 310,007, including N = 35 from Ukraine). We then down-sampled the number of sequences in all location to 12, except for the countries with top-10 number of Delta variant cases in the world (USA, UK, France, Germany, Denmark, India, Canada, Turkey, Sweden, and Switzerland)^42^, countries that share border with Ukraine (Russia, Moldova, Romania, Hungary, Poland, Slovakia, Belarus), and other post-Soviet countries, including the three Baltic nations (Estonia, Latvia, Lithuania), Georgia, and Uzbekistan (no Delta sequences were available from the other post-Soviet countries in Central Asia and Transcaucasia); we included 36 sequences from each of these locations in the analysis. We then combined the down-sampled dataset and the 21 Delta sequences produced in this study in August 2021 and applied the *mugration* model implemented in TreeTime^43^ to estimate the number and originating locations of Delta variant introductions in Ukraine. The *mugration* model assumes countries of origin as discreet states and considers the virus spread between these states as a general time-reversible process.

## Supplementary Information

Supplementary Information 1.

Supplementary Information 2.

Supplementary Information 3.

Supplementary Information 4.

Supplementary Information 5.

Supplementary Information 6.

## Data Availability

Sequence accession numbers for the assembled/consensus genomes in GISAID are as follows: EPI_ISL_5852904, EPI_ISL_5852905, EPI_ISL_5852906, EPI_ISL_5852907, EPI_ISL_5852908, EPI_ISL_5852909, EPI_ISL_5852910, EPI_ISL_5852911, EPI_ISL_5852912, EPI_ISL_5852913, EPI_ISL_5852914, EPI_ISL_5852915, EPI_ISL_5852916, EPI_ISL_5852917, EPI_ISL_5852918, EPI_ISL_5852919, EPI_ISL_5852920, EPI_ISL_5852921, EPI_ISL_5852922, EPI_ISL_5852923, EPI_ISL_5852924, EPI_ISL_5852925, EPI_ISL_5852926, EPI_ISL_5859548, EPI_ISL_9662995, EPI_ISL_9662954, EPI_ISL_9662969, EPI_ISL_9662985, EPI_ISL_9662986, EPI_ISL_9662982, EPI_ISL_9662988, EPI_ISL_9662989, EPI_ISL_9662962, EPI_ISL_9662961, EPI_ISL_9662994, EPI_ISL_9662970, EPI_ISL_9662978, EPI_ISL_9662971, EPI_ISL_9662979, EPI_ISL_9662987, EPI_ISL_9662981, EPI_ISL_9662956, EPI_ISL_9662966, EPI_ISL_9663021, EPI_ISL_9663022, EPI_ISL_9663023, EPI_ISL_9663024, EPI_ISL_9663025, EPI_ISL_9663026, EPI_ISL_9663027, EPI_ISL_9663028, EPI_ISL_9663014, EPI_ISL_9663015, EPI_ISL_9663016, EPI_ISL_9663017, EPI_ISL_9663018, EPI_ISL_9663019, EPI_ISL_9663020, EPI_ISL_9663004, EPI_ISL_9663005, EPI_ISL_9663006, EPI_ISL_9663007, EPI_ISL_9663008, EPI_ISL_9663009, EPI_ISL_9663010, EPI_ISL_9663011, EPI_ISL_9663012, EPI_ISL_9663013, EPI_ISL_9662976, EPI_ISL_9662996, EPI_ISL_9662997, EPI_ISL_9662998, EPI_ISL_9662999, EPI_ISL_9663000, EPI_ISL_9663002, EPI_ISL_9662955, EPI_ISL_9663003, EPI_ISL_9663001, EPI_ISL_9662990, EPI_ISL_9662984, EPI_ISL_9662991, EPI_ISL_9662993, EPI_ISL_9662992, EPI_ISL_9662967, EPI_ISL_9662983, EPI_ISL_9662977, EPI_ISL_9662957, EPI_ISL_9662958, EPI_ISL_9662972, EPI_ISL_9662980, EPI_ISL_9662960, EPI_ISL_9662959, EPI_ISL_9662968, EPI_ISL_9662963, EPI_ISL_9662973, EPI_ISL_9663029, EPI_ISL_9663031, EPI_ISL_9663032, EPI_ISL_9663030, EPI_ISL_9662964, EPI_ISL_9662965, EPI_ISL_9662974, EPI_ISL_9662975.
