# Supplementary figures and images for: Tracking SARS-COV-2 variants using Nanopore sequencing in Ukraine in 2021
Source: Sci Rep. 2022 Sep 21;12:15749. doi: 10.1038/s41598-022-19414-y (PMC9491264; doi:10.1038/s41598-022-19414-y)

**Figure S1.** The SARS-CoV-2 predominant lineages distribution in Ukraine.

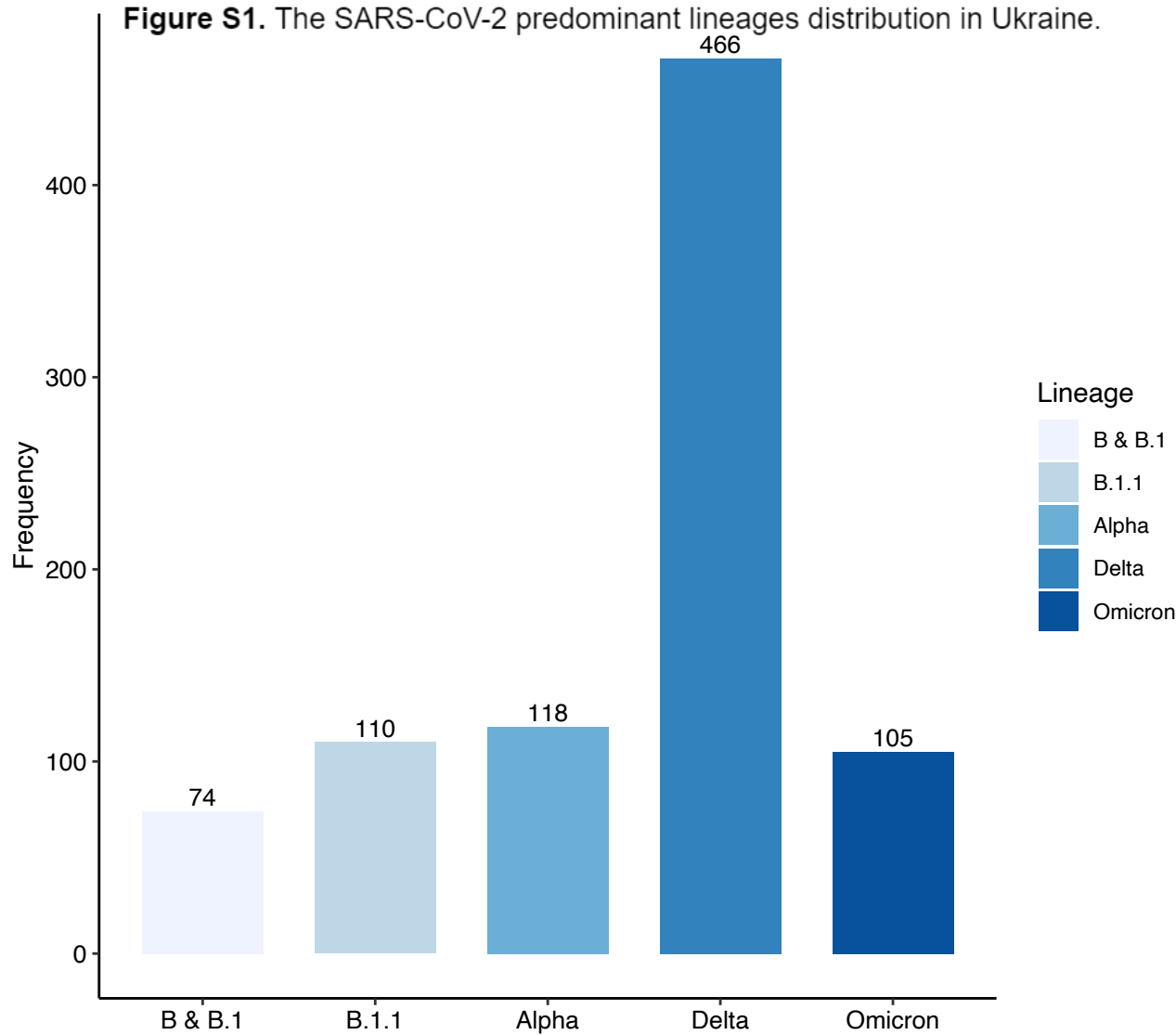

Supplement: Supplementary file 1 — Supplementary Information 1. [file 41598_2022_19414_MOESM1_ESM.pdf]
