# Supplementary material for: Tracking SARS-COV-2 variants using Nanopore sequencing in Ukraine in 2021
Source: Sci Rep. 2022 Sep 21;12:15749. doi: 10.1038/s41598-022-19414-y (PMC9491264; doi:10.1038/s41598-022-19414-y)

Figure S2. The number of SARS-CoV-2 sequences and COVID-19 cases from each epidemic wave in Ukraine. The sequence data represent approximately 0.02% of the total COVID-19 confirmed cases.

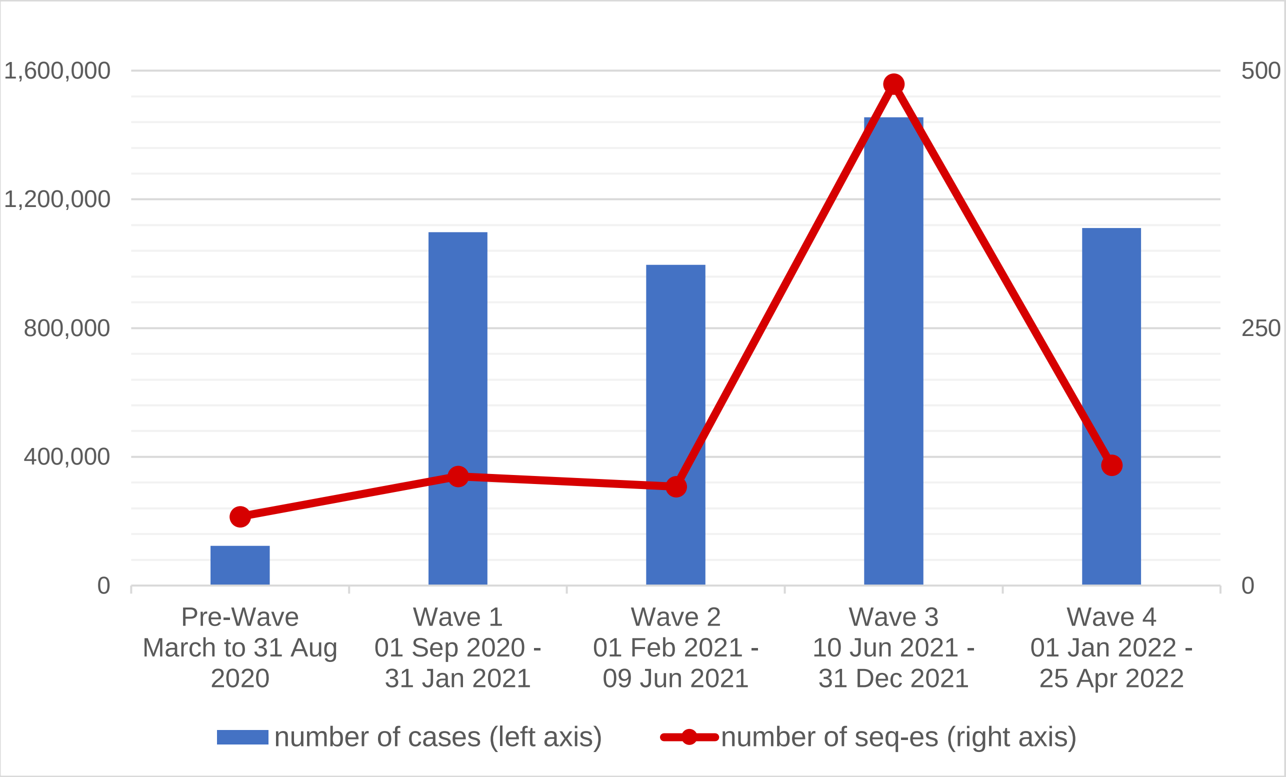

Supplement: Supplementary file 2 — Supplementary Information 2. [file 41598_2022_19414_MOESM2_ESM.pdf]
