# Supplementary material for: Tracking SARS-COV-2 variants using Nanopore sequencing in Ukraine in 2021
Source: Sci Rep. 2022 Sep 21;12:15749. doi: 10.1038/s41598-022-19414-y (PMC9491264; doi:10.1038/s41598-022-19414-y)

Figure S3. Phylogenetic tree of the SARS-CoV-2 Delta variant sequences circulating in summer 2021 and selected for phylogeographic analysis. Red circles indicate the SARS-C0V-2 Delta genomes from Ukraine.

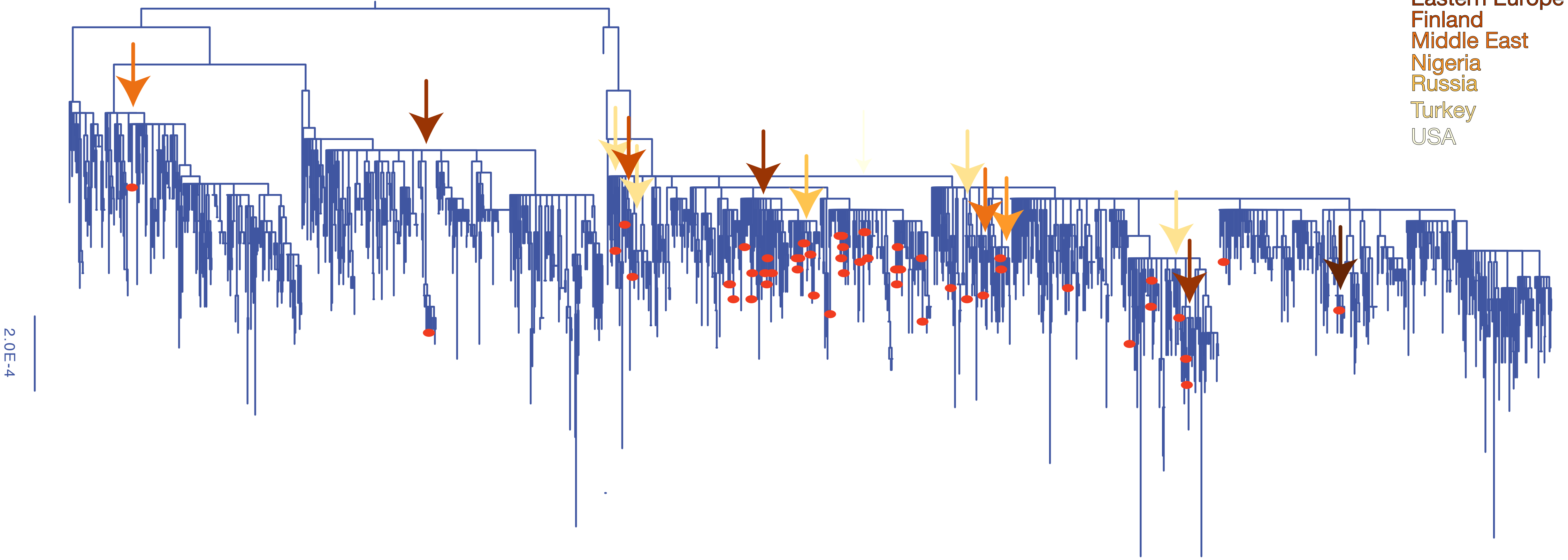

Supplement: Supplementary file 3 — Supplementary Information 3. [file 41598_2022_19414_MOESM3_ESM.pdf]
