# Supplementary material for: Tracking SARS-COV-2 variants using Nanopore sequencing in Ukraine in 2021
Source: Sci Rep. 2022 Sep 21;12:15749. doi: 10.1038/s41598-022-19414-y (PMC9491264; doi:10.1038/s41598-022-19414-y)
Supplement: Supplementary file 4 — Supplementary Information 4. [file 41598_2022_19414_MOESM4_ESM.pdf]

Table S4. Acknowledgement GISAID table.
We gratefully acknowledge the following Authors from the Originating laboratories responsible for obtaining the specimens, as well as the Submitting laboratories where the genome data were generated and shared via GISAID, on which this research is based.
All Submitters of data may be contacted directly via www.gisaid.org

Accession ID	Originating Laboratory	Submitting Laboratory	Authors
EPI_ISL_1298483, EPI_ISL_1298488	Bogorodchany CRH	The Institute of Molecular Biology and Genetics of NASU	M.Tukalo et al.
EPI_ISL_1298474	Burshtyn CCH	The Institute of Molecular Biology and Genetics of NASU	M.Tukalo et al.
EPI_ISL_576146, EPI_ISL_576147, EPI_ISL_576148, EPI_ISL_576149	Department of Respiratory & Other Viral Infections of L.V. Gromashevsky Institute of Epidemiology & Infectious Diseases NAMS of Ukraine	Department of Respiratory & Other Viral Infections of L.V. Gromashevsky Institute of Epidemiology & Infectious Diseases NAMS of Ukraine, JSC "Farmak"	Alla Mironenko; Andriy Goy; Ihor Kravchuk; Larysa Radchenko; Liudmyla Bolotova; Nataliia Teteriuk
EPI_ISL_4260382, EPI_ISL_4260384, EPI_ISL_4260386, EPI_ISL_4260388, EPI_ISL_4260390, EPI_ISL_4260392, EPI_ISL_4260393, EPI_ISL_4260395, EPI_ISL_4260396, EPI_ISL_4260398, EPI_ISL_4260400, EPI_ISL_4260402, EPI_ISL_4260404, EPI_ISL_4260406, EPI_ISL_4260408, EPI_ISL_4260410, EPI_ISL_4260411, EPI_ISL_4260413, EPI_ISL_4260414, EPI_ISL_4260416, EPI_ISL_4260418, EPI_ISL_4260420, EPI_ISL_4260424, EPI_ISL_4260427, EPI_ISL_4260429, EPI_ISL_4260430, EPI_ISL_4260432, EPI_ISL_4260433, EPI_ISL_4260435, EPI_ISL_4260436, EPI_ISL_4260438, EPI_ISL_4260440, EPI_ISL_4260442, EPI_ISL_4260444, EPI_ISL_4260445, EPI_ISL_4260449, EPI_ISL_4260450			
see above	Department of Respiratory and other Viral Infections of L.V. Gromashevsky Institute of Epidemiology and Infectious Diseases NAMS of Ukarine	CNR Virus des Infections Respiratoires - France SUD	Alla Mironenko; Antonin Bal; Bruno Lina; Gregory Destras; Gwendolyne Burfin; Hadrien Regue; Larysa Radchenko; Laurence Josset; Martine Valette; Nataliia Teteriuk; Quentin Semanas
EPI_ISL_582509	Department of Respiratory and other Viral Infections of L.V.Gromashevsky Institute of Epidemiology & Infectious Diseases NAMS of Ukraine	Department of Respiratory and other Viral Infections of L.V.Gromashevsky Institute of Epidemiology & Infectious Diseases NAMS of Ukraine, JSC "Farmak"	Alla Mironenko; Andriy Goy; Ihor Kravchuk; Larysa Radchenko; Ludmyla Bolotova; Nataliia Teteriuk
EPI_ISL_582510	Department of Respiratory and other Viral Infections of L.V.Gromashevsky Institute of Epidemiology & Infectious Diseases NAMS of Ukraine	Department of Respiratory and other Viral Infections of L.V.Gromashevsky Institute of Epidemiology & Infectious Diseases NAMS of Ukraine, JSC "Farmak"	Alla Mironenko; Andriy Goy; Ihor Kravchuk; Larysa Radchenko; Ludmyla Bolotova; Nataliia Teteriuk
EPI_ISL_582511, EPI_ISL_582512, EPI_ISL_582513, EPI_ISL_654819, EPI_ISL_654820, EPI_ISL_979970, EPI_ISL_979971, EPI_ISL_979972, EPI_ISL_979973, EPI_ISL_1121990, EPI_ISL_1121991, EPI_ISL_1121992, EPI_ISL_1122014, EPI_ISL_1315427, EPI_ISL_1315475, EPI_ISL_1315476, EPI_ISL_1315477, EPI_ISL_1315478			
see above	Department of Respiratory and other Viral Infections of L.V.Gromashevsky Institute of Epidemiology & Infectious Diseases NAMS of Ukraine	Department of Respiratory and other Viral Infections of L.V.Gromashevsky Institute of Epidemiology & Infectious Diseases NAMS of Ukraine, JSC "Farmak"	Alla Mironenko; Andriy Goy; Ihor Kravchuk; Larysa Radchenko; Ludmyla Bolotova; Nataliia Teteriuk
EPI_ISL_654818	Department of Respiratory and other Viral Infections of L.V.Gromashevsky Institute of Epidemiology & Infectious Diseases NAMS of Ukraine	Department of Respiratory and other Viral Infections of L.V.Gromashevsky Institute of Epidemiology & Infectious Diseases NAMS of Ukraine, JSC "Farmak"	Alla Mironenko; Andriy Goy; Ihor Kravchuk; Larysa Radchenko; Ludmyla Bolotova; Nataliia Teteriuk
EPI_ISL_3527830, EPI_ISL_5881144	Diagen	Diagen	Koliada O; Koliada et al
EPI_ISL_3253254, EPI_ISL_3253255, EPI_ISL_3253256, EPI_ISL_3253257, EPI_ISL_3253258, EPI_ISL_3253259, EPI_ISL_3253260, EPI_ISL_3253261, EPI_ISL_3253262, EPI_ISL_3253263, EPI_ISL_3253264, EPI_ISL_3253265, EPI_ISL_3253266, EPI_ISL_3253267, EPI_ISL_3253268, EPI_ISL_3253269, EPI_ISL_3253270, EPI_ISL_3253271, EPI_ISL_3253272, EPI_ISL_3253273, EPI_ISL_3253274, EPI_ISL_3253275, EPI_ISL_3253276, EPI_ISL_3253277, EPI_ISL_3253278, EPI_ISL_3253279, EPI_ISL_3253280, EPI_ISL_3253281, EPI_ISL_3253282, EPI_ISL_3253283, EPI_ISL_3253284, EPI_ISL_3253285, EPI_ISL_3253286, EPI_ISL_3253287, EPI_ISL_3253288, EPI_ISL_3253289, EPI_ISL_3253290, EPI_ISL_3253291, EPI_ISL_3253292, EPI_ISL_3253293, EPI_ISL_3253294, EPI_ISL_3253295, EPI_ISL_3253296, EPI_ISL_3253297, EPI_ISL_3314947, EPI_ISL_3314948, EPI_ISL_3314949			
see above	Dutch COVID-19 response team	National Institute for Public Health and the Environment (RIVM)	Adam Meijer; AnneMarie van den Brandt; Annelies Kroneman; Bas van der Veer; Chantal Reusken; Dennis Schmitz; Dirk Eggink; Euníce Then; Florian Zwagemaker; Harry Vennema; Jeroen Cremer; Karim Hajji; Kim Freriks; Lisa Wijsman; Lynn Aarts; Melissa van Tuil; Ryanne Jaarsma; Sanne Bos; Sharon van den Brink; on behalf of the national COVID-19 response team
EPI_ISL_1298476, EPI_ISL_1298477, EPI_ISL_1298481	Ivano-Frankivsk CCH#1	The Institute of Molecular Biology and Genetics of NASU	M.Tukalo et al.
EPI_ISL_1298487	Kalush CRH	The Institute of Molecular Biology and Genetics of NASU	M.Tukalo et al.
EPI_ISL_6359793	Klinisch Laboratorium ZNA	Klinisch Laboratorium ZNA	Verstrepen et al.
EPI_ISL_1298475, EPI_ISL_1298478, EPI_ISL_1298479, EPI_ISL_1298485, EPI_ISL_1298486	Kosov CRH	The Institute of Molecular Biology and Genetics of NASU	M.Tukalo et al.
EPI_ISL_5852904, EPI_ISL_5852905, EPI_ISL_5852906, EPI_ISL_5852907, EPI_ISL_5852908, EPI_ISL_5852909, EPI_ISL_5852910, EPI_ISL_5852911, EPI_ISL_5852912, EPI_ISL_5852913, EPI_ISL_5852914, EPI_ISL_5852915, EPI_ISL_5852916, EPI_ISL_5852917, EPI_ISL_5852918, EPI_ISL_5852919, EPI_ISL_5852920, EPI_ISL_5852921, EPI_ISL_5852922, EPI_ISL_5852923, EPI_ISL_5852924, EPI_ISL_5852925, EPI_ISL_5852926, EPI_ISL_5859548			
see above	L.V. Gromashevsky Institute of Epidemiology and Infectious Diseases of the NAMS of Ukraine	L.V. Gromashevsky Institute of Epidemiology and Infectious Diseases of the NAMS of Ukraine	Alla M. Scherbinska; Anna Yakovleva; Eric Bortz; Ganna Kovalenko; Ian Goodfellow; Joel O. Wertheim; Luke Meredith; Mariia G. Liulchuk; Matthew Redlinger; Tetyana I. Vasylyeva; Viktoria I. Zadorozhna
EPI_ISL_1298480, EPI_ISL_1298484	Nadvirna CRH	The Institute of Molecular Biology and Genetics of NASU	M.Tukalo et al.
EPI_ISL_512597, EPI_ISL_512598, EPI_ISL_512599, EPI_ISL_512600, EPI_ISL_512601, EPI_ISL_512602, EPI_ISL_512603, EPI_ISL_512604, EPI_ISL_512605, EPI_ISL_512606, EPI_ISL_512607, EPI_ISL_512608, EPI_ISL_512609, EPI_ISL_512610, EPI_ISL_512611, EPI_ISL_512612, EPI_ISL_512613, EPI_ISL_512614, EPI_ISL_512615, EPI_ISL_512616, EPI_ISL_512617, EPI_ISL_512618, EPI_ISL_512619, EPI_ISL_512620, EPI_ISL_512621, EPI_ISL_512622, EPI_ISL_512623, EPI_ISL_512624, EPI_ISL_512625, EPI_ISL_512626, EPI_ISL_512627, EPI_ISL_512628, EPI_ISL_512629, EPI_ISL_512630, EPI_ISL_512631, EPI_ISL_512632, EPI_ISL_512633, EPI_ISL_512634, EPI_ISL_512635, EPI_ISL_512636, EPI_ISL_512637, EPI_ISL_512639, EPI_ISL_512640, EPI_ISL_512641, EPI_ISL_512642, EPI_ISL_512643, EPI_ISL_512644, EPI_ISL_512810			
see above	National Laboratory for Influenza/Virology reference laboratory, Public Health Center of the Ministry of Health of Ukraine	Respiratory Virus Unit, Microbiology Services Colindale, Public Health England	Dr. Iryna Demchyshyna; PHE Covid Sequencing Team
EPI_ISL_4440036, EPI_ISL_4440039, EPI_ISL_4440042, EPI_ISL_4440044, EPI_ISL_4440046, EPI_ISL_4440048, EPI_ISL_4440053, EPI_ISL_4440055, EPI_ISL_4440057, EPI_ISL_4440060, EPI_ISL_4440062, EPI_ISL_4440064, EPI_ISL_4440066, EPI_ISL_4440068, EPI_ISL_4440071, EPI_ISL_4440073, EPI_ISL_4440075, EPI_ISL_4440078, EPI_ISL_4440080, EPI_ISL_4440082, EPI_ISL_4440084, EPI_ISL_4440087, EPI_ISL_4440089, EPI_ISL_4440092, EPI_ISL_4440093, EPI_ISL_4440095, EPI_ISL_4440097, EPI_ISL_4440099, EPI_ISL_4440101, EPI_ISL_4440103			
see above	Public Health Center of MHU	The Institute of Molecular Biology and Genetics of NASU	M.Tukalo et al.
EPI_ISL_1112293, EPI_ISL_1112294, EPI_ISL_1112295, EPI_ISL_1112296, EPI_ISL_1112297, EPI_ISL_1112298, EPI_ISL_1112299, EPI_ISL_1112300, EPI_ISL_1112301, EPI_ISL_1112302, EPI_ISL_1112303, EPI_ISL_1112304, EPI_ISL_1112305, EPI_ISL_1112306, EPI_ISL_1112307, EPI_ISL_1112308, EPI_ISL_1112309, EPI_ISL_1112310, EPI_ISL_1112311, EPI_ISL_1112312, EPI_ISL_1112313, EPI_ISL_1112314, EPI_ISL_1112315, EPI_ISL_1112316, EPI_ISL_1112317, EPI_ISL_1112318, EPI_ISL_1112319, EPI_ISL_1112320, EPI_ISL_1112321, EPI_ISL_1112322, EPI_ISL_1112323, EPI_ISL_1112324, EPI_ISL_1112325, EPI_ISL_1112326, EPI_ISL_1112327, EPI_ISL_1112328, EPI_ISL_1112329, EPI_ISL_1112330, EPI_ISL_1112331, EPI_ISL_1112332, EPI_ISL_1112333, EPI_ISL_1112334, EPI_ISL_1112335, EPI_ISL_1112336, EPI_ISL_1112337, EPI_ISL_1112338, EPI_ISL_1112339, EPI_ISL_1112753			
see above	Public Health Center of Ukraine	Charité Universitätsmedizin Berlin, Institute of Virology	Barbara Mühlemann; Christian Drosten; Ihor Kuzin; Iryna Demchyshyna; Julia Schneider; Jörn Beheim-Schwarzbach; Liudmyla Chernenko; Roman Rodyna; Talitha Veith; Terry Jones; Victor M Corman
EPI_ISL_2934571, EPI_ISL_2934572, EPI_ISL_2934575, EPI_ISL_2966511, EPI_ISL_2966513	SE Kyiv CDC MHU	The Institute of Molecular Biology and Genetics of NASU	M.Tukalo et al.
EPI_ISL_2934569, EPI_ISL_2934570, EPI_ISL_2934573, EPI_ISL_2934574, EPI_ISL_2966512	SECP MHU	The Institute of Molecular Biology and Genetics of NASU	M.Tukalo et al.
EPI_ISL_3262118, EPI_ISL_3262119, EPI_ISL_3262120, EPI_ISL_3262121, EPI_ISL_3262122, EPI_ISL_3262123, EPI_ISL_3262124, EPI_ISL_3262125, EPI_ISL_3262126, EPI_ISL_3262127, EPI_ISL_3262128, EPI_ISL_3262129, EPI_ISL_3262130, EPI_ISL_3262131, EPI_ISL_3262132, EPI_ISL_3262133, EPI_ISL_3262134, EPI_ISL_3262135, EPI_ISL_3262136, EPI_ISL_3262137, EPI_ISL_3262138, EPI_ISL_5633415, EPI_ISL_5633416, EPI_ISL_5633417, EPI_ISL_5633418, EPI_ISL_5633419, EPI_ISL_5633420, EPI_ISL_5633421, EPI_ISL_5633422, EPI_ISL_5633423, EPI_ISL_5633424, EPI_ISL_5633425, EPI_ISL_5633426, EPI_ISL_5633427, EPI_ISL_5633428, EPI_ISL_5633429, EPI_ISL_5633430, EPI_ISL_5633431, EPI_ISL_5633432, EPI_ISL_5633433, EPI_ISL_5633434, EPI_ISL_5633435, EPI_ISL_5633436, EPI_ISL_5633437, EPI_ISL_5633438, EPI_ISL_5633439, EPI_ISL_5633440, EPI_ISL_5881037, EPI_ISL_5881038, EPI_ISL_5881039, EPI_ISL_5881040, EPI_ISL_5881041, EPI_ISL_5881042, EPI_ISL_5881043, EPI_ISL_5881044, EPI_ISL_5881045, EPI_ISL_5881046, EPI_ISL_5881047, EPI_ISL_5881048, EPI_ISL_5881049, EPI_ISL_5881050, EPI_ISL_5881051, EPI_ISL_5881052, EPI_ISL_5881053, EPI_ISL_5881054, EPI_ISL_5881055, EPI_ISL_5881056, EPI_ISL_5881057, EPI_ISL_5881058, EPI_ISL_5881059, EPI_ISL_5881060, EPI_ISL_5881061, EPI_ISL_5881062, EPI_ISL_5881063, EPI_ISL_5881064, EPI_ISL_5881065, EPI_ISL_5881066, EPI_ISL_5881067, EPI_ISL_5881068, EPI_ISL_5881069, EPI_ISL_5881070, EPI_ISL_5881071, EPI_ISL_5884463, EPI_ISL_6280195, EPI_ISL_6280202, EPI_ISL_6280207, EPI_ISL_6280220, EPI_ISL_6280226, EPI_ISL_6280232, EPI_ISL_6280241, EPI_ISL_6280248, EPI_ISL_6280254, EPI_ISL_6280262, EPI_ISL_6280271, EPI_ISL_6280281, EPI_ISL_6280290, EPI_ISL_6280301, EPI_ISL_6280305, EPI_ISL_6280313, EPI_ISL_6280318, EPI_ISL_6280325, EPI_ISL_6280331, EPI_ISL_6280341, EPI_ISL_6280350, EPI_ISL_6280358, EPI_ISL_6280366, EPI_ISL_6280374, EPI_ISL_6280379, EPI_ISL_6280383, EPI_ISL_6280391, EPI_ISL_6280399, EPI_ISL_6280405, EPI_ISL_6280413, EPI_ISL_6280422, EPI_ISL_6280428, EPI_ISL_6700698, EPI_ISL_6700699, EPI_ISL_6700700, EPI_ISL_6700701, EPI_ISL_6700702, EPI_ISL_6700703, EPI_ISL_6700704, EPI_ISL_6700710, EPI_ISL_6700716, EPI_ISL_6700723, EPI_ISL_6700731, EPI_ISL_6700739, EPI_ISL_6700746, EPI_ISL_6700748, EPI_ISL_6700753, EPI_ISL_6700763, EPI_ISL_6700768, EPI_ISL_6700776, EPI_ISL_6700785, EPI_ISL_6700793, EPI_ISL_6700794, EPI_ISL_6700795, EPI_ISL_6700796, EPI_ISL_6700797, EPI_ISL_6700798, EPI_ISL_6700799, EPI_ISL_6700800, EPI_ISL_6700801, EPI_ISL_6700802, EPI_ISL_6700803, EPI_ISL_6700804, EPI_ISL_6700805, EPI_ISL_6700806, EPI_ISL_6700807, EPI_ISL_6700808, EPI_ISL_6700809, EPI_ISL_6705650			
see above	SI «Public Health Center of MHU»	The Institute of Molecular Biology and Genetics of NASU	M.Tukalo et al.
EPI_ISL_1495111, EPI_ISL_1495112, EPI_ISL_1495113, EPI_ISL_1495114, EPI_ISL_1495115, EPI_ISL_1495116, EPI_ISL_1495117, EPI_ISL_1495118, EPI_ISL_1495119, EPI_ISL_1495120, EPI_ISL_1495121, EPI_ISL_1495122, EPI_ISL_1495123, EPI_ISL_1495124, EPI_ISL_1495125, EPI_ISL_1495126, EPI_ISL_1495127, EPI_ISL_1495128, EPI_ISL_1495129, EPI_ISL_1495130, EPI_ISL_1495131, EPI_ISL_1495132, EPI_ISL_1495133, EPI_ISL_1495134, EPI_ISL_1495135, EPI_ISL_1495136, EPI_ISL_1495137, EPI_ISL_1495138, EPI_ISL_1495139, EPI_ISL_1495140, EPI_ISL_1495141, EPI_ISL_1495142, EPI_ISL_1495143			
see above	State Institution «Public Health Center of Ministry of Health of Ukraine»	Robert Koch Institute, ZBS1 Highly Pathogenic Viruses, Berlin, Germany	Andreas Nitsche; Annika Brinkmann; Iryna Demchyshyna; Janine Michel; Liudmyla Chernenko; Roman Rodyna; Steven Uddin
EPI_ISL_1298482	Tysmenytsia CH	The Institute of Molecular Biology and Genetics of NASU	M.Tukalo et al.

We gratefully acknowledge the following Authors from the Originating laboratories responsible for obtaining the specimens, as well as the Submitting laboratories where the genome data were generated and shared via GISAID, on which this research is based.

All Submitters of data may be contacted directly via www.gisaid.org

**Accession ID**	**Originating Laboratory**	**Submitting Laboratory**	**Authors**
EPI_ISL_7398746	"Dila" medical laboratory	"Dila" medical laboratory	Kostiantyn Nekrasov
EPI_ISL_8917339	2021-12-04	Jiaxing Center for Disease Control and Prevention	Guoying Zhu; Ping Li; Shencong Lv; Yin Song; Yong Yan; Zhongwen Chen
EPI_ISL_8142634	AZ St.Jan Brugge-Oostende	AZ St.Jan Brugge-Oostende	Jorn Hellemans; Marie Madeleine Chabert-Consen; Marijke Reynders; Patrick Descheemaeker; Sofie Mahboob
EPI_ISL_9663029, EPI_ISL_9663030, EPI_ISL_9663031, EPI_ISL_9663032	Center for Family Medicine of Ukrainian Medical Diagnostic Center	L.V. Gromashevsky Institute of Epidemiology and Infectious Diseases of the NAMS of Ukraine	Alla M. Scherbinska; Anna Yakovleva; Eric Bortz; Ganna Kovalenko; Ian Goodfellow; Joel O. Wertheim; Luke Meredith; Mariia G. Liulchuk; Matthew Redlinger; Tetyana I. Vasylyeva; Viktoria I. Zadorozhna
EPI_ISL_12436296, EPI_ISL_12436297, EPI_ISL_12436298, EPI_ISL_12436299, EPI_ISL_12436300, EPI_ISL_12436322, EPI_ISL_12436324, EPI_ISL_12436325, EPI_ISL_12546574, EPI_ISL_12546614, EPI_ISL_12546615, EPI_ISL_12546616, EPI_ISL_12546617, EPI_ISL_12546618, EPI_ISL_12546619, EPI_ISL_12546632			
see above	Department of Respiratory and other Viral Infections of L.V. Gromashevsky Institute of Epidemiology & Infectious Diseases NAMS of Ukraine	Department of Respiratory and other Viral Infections of L.V. Gromashevsky Institute of Epidemiology & Infectious Diseases NAMS of Ukraine, JSC Farmak	Alla Mironenko; Andriy Goy; Ihor Kravchuk; Larysa Radchenko; Nataliia Teteriuk; Pydiura Mykola
EPI_ISL_12267825, EPI_ISL_12267826, EPI_ISL_12267827, EPI_ISL_12267828, EPI_ISL_12267829, EPI_ISL_12267830, EPI_ISL_12267831, EPI_ISL_12267832			
see above	Department of Respiratory and other Viral Infections of L.V. Gromashevsky Institute of Epidemiology and Infectious Diseases NAMS of Ukarine, 33	CNR Virus des Infections Respiratoires - France SUD	Alla Mironenko; Antonin Bal; Bruno Lina; Bruno Simon; Gregory Destras; Gwendolyne Burfin; Hadrien Regue; Larysa Radchenko; Laurence Josset; Martine Valette; Nataliia Teteriuk; Quentin Semanas; Theophile Boyer
EPI_ISL_7395070, EPI_ISL_7396045, EPI_ISL_7396713, EPI_ISL_7397609, EPI_ISL_7755847, EPI_ISL_7756082, EPI_ISL_7756831, EPI_ISL_7757043, EPI_ISL_7757243, EPI_ISL_7757467, EPI_ISL_7757655, EPI_ISL_7758462, EPI_ISL_10047226, EPI_ISL_10047227, EPI_ISL_10047228, EPI_ISL_11866940, EPI_ISL_11866941, EPI_ISL_11866942, EPI_ISL_11866943, EPI_ISL_11866944, EPI_ISL_11866945, EPI_ISL_12063453, EPI_ISL_12063603, EPI_ISL_12063604, EPI_ISL_12249368, EPI_ISL_12249372, EPI_ISL_12249374, EPI_ISL_12249377, EPI_ISL_12249380, EPI_ISL_12249423			
see above	Department of Respiratory and other Viral Infections of L.V.Gromashevsky Institute of Epidemiology & Infectious Diseases NAMS of Ukraine	Department of Respiratory and other Viral Infections of L.V.Gromashevsky Institute of Epidemiology & Infectious Diseases NAMS of Ukraine, JSC "Farmak"	Alla Mironenko; Andriy Goy; Ihor Kravchuk; Larysa Radchenko; Mykola Pydiura; Nataliia Teteriuk; Pydiura Mykola
EPI_ISL_10717566, EPI_ISL_10717567, EPI_ISL_10717568, EPI_ISL_10717569, EPI_ISL_10717570, EPI_ISL_10717571, EPI_ISL_10717572, EPI_ISL_10717573			
see above	L.V.GROMASHEVSKY	CNR Virus des Infections Respiratoires - France SUD	Antonin Bal; Bruno Lina; Bruno Simon; Gregory Destras; Gwendolyne Burfin; Hadrien Regue; Laurence Josset; Martine Valette; Quentin Semanas; Theophile Boyer
EPI_ISL_7987086	Medical laboratory "Dila"	Medical laboratory "Dila"	B.Shpyga; D.Negrulia; K.Nekrasov; Y.Natalych
EPI_ISL_9479659	Platform BIS UZA/UAntwerpen	Labo Klinische Biologie, UZA	Basil Britto Xavier; Christine Lammens; Herman Goossens; Ines Verbesselt; Jasmine Coppens; Kathleen Holemans; Marie Le Mercier; Veerle Matheeussen
EPI_ISL_7967972, EPI_ISL_7967973, EPI_ISL_7967974, EPI_ISL_7967975, EPI_ISL_7967976, EPI_ISL_7967977, EPI_ISL_7967978, EPI_ISL_7967979, EPI_ISL_7967980, EPI_ISL_7967981, EPI_ISL_7967982, EPI_ISL_7967983, EPI_ISL_7967984, EPI_ISL_7967985, EPI_ISL_7967986, EPI_ISL_7967987, EPI_ISL_7967988, EPI_ISL_7967989, EPI_ISL_7967990, EPI_ISL_7967991, EPI_ISL_7967992, EPI_ISL_7967993, EPI_ISL_7967994, EPI_ISL_7967995, EPI_ISL_7967996, EPI_ISL_7967997, EPI_ISL_7967998, EPI_ISL_7967999, EPI_ISL_7968000, EPI_ISL_7968001, EPI_ISL_7968002, EPI_ISL_7968003, EPI_ISL_7968004, EPI_ISL_7968005, EPI_ISL_7968006, EPI_ISL_7968007, EPI_ISL_7968008, EPI_ISL_7968009, EPI_ISL_7968010, EPI_ISL_7968011, EPI_ISL_7968012, EPI_ISL_7968013, EPI_ISL_8645799, EPI_ISL_8645800, EPI_ISL_8645801, EPI_ISL_8645802, EPI_ISL_8645803, EPI_ISL_8645804, EPI_ISL_8645805, EPI_ISL_8645806, EPI_ISL_8645807, EPI_ISL_8645808, EPI_ISL_8645809, EPI_ISL_8645810, EPI_ISL_8645811, EPI_ISL_8645812, EPI_ISL_8645813, EPI_ISL_8645814, EPI_ISL_8645815, EPI_ISL_8645816, EPI_ISL_8645820, EPI_ISL_8645821, EPI_ISL_8645822, EPI_ISL_9859601, EPI_ISL_9859602, EPI_ISL_9859603, EPI_ISL_9859604, EPI_ISL_9859605, EPI_ISL_9859606, EPI_ISL_9859607, EPI_ISL_9859608, EPI_ISL_9859609, EPI_ISL_9859610, EPI_ISL_9859611, EPI_ISL_9859612, EPI_ISL_9859613, EPI_ISL_9859614, EPI_ISL_9859615, EPI_ISL_9859616, EPI_ISL_9859617, EPI_ISL_9859618, EPI_ISL_9859619, EPI_ISL_9859620, EPI_ISL_9859621, EPI_ISL_9859622, EPI_ISL_9859623, EPI_ISL_9859624, EPI_ISL_9859625, EPI_ISL_9859626, EPI_ISL_9859627, EPI_ISL_9859628, EPI_ISL_9859629, EPI_ISL_9859631, EPI_ISL_9859632, EPI_ISL_9859633, EPI_ISL_9859634, EPI_ISL_9859635, EPI_ISL_9859636, EPI_ISL_11438686, EPI_ISL_11438687, EPI_ISL_11438688, EPI_ISL_11438689, EPI_ISL_11438690, EPI_ISL_11438691, EPI_ISL_11438692, EPI_ISL_11438693, EPI_ISL_11438694, EPI_ISL_11438695, EPI_ISL_11438696, EPI_ISL_11438697, EPI_ISL_11438698, EPI_ISL_11438699, EPI_ISL_11438700, EPI_ISL_11438701, EPI_ISL_11438702, EPI_ISL_11438703, EPI_ISL_11438704, EPI_ISL_11438705, EPI_ISL_11438706, EPI_ISL_11438707, EPI_ISL_11438708, EPI_ISL_11438709, EPI_ISL_11438710, EPI_ISL_11438711, EPI_ISL_11438712, EPI_ISL_11438713, EPI_ISL_11438714, EPI_ISL_11438715, EPI_ISL_11438716, EPI_ISL_11438717, EPI_ISL_11438718, EPI_ISL_11438719, EPI_ISL_11438720, EPI_ISL_11438721, EPI_ISL_11438722			
see above	SI «Public Health Center of MHU»	The Institute of Molecular Biology and Genetics of NASU	M.Tukalo et al.
EPI_ISL_9662954, EPI_ISL_9662955, EPI_ISL_9662956, EPI_ISL_9662957, EPI_ISL_9662958, EPI_ISL_9662959, EPI_ISL_9662960, EPI_ISL_9662961, EPI_ISL_9662962, EPI_ISL_9662963, EPI_ISL_9662964, EPI_ISL_9662965, EPI_ISL_9662966, EPI_ISL_9662967, EPI_ISL_9662968, EPI_ISL_9662969, EPI_ISL_9662970, EPI_ISL_9662971, EPI_ISL_9662972, EPI_ISL_9662973, EPI_ISL_9662974, EPI_ISL_9662975, EPI_ISL_9662976, EPI_ISL_9662977, EPI_ISL_9662978, EPI_ISL_9662979, EPI_ISL_9662980, EPI_ISL_9662981, EPI_ISL_9662982, EPI_ISL_9662983, EPI_ISL_9662984, EPI_ISL_9662985, EPI_ISL_9662986, EPI_ISL_9662987, EPI_ISL_9662988, EPI_ISL_9662989, EPI_ISL_9662990, EPI_ISL_9662991, EPI_ISL_9662992, EPI_ISL_9662993, EPI_ISL_9662994, EPI_ISL_9662995, EPI_ISL_9662996, EPI_ISL_9662997, EPI_ISL_9662998, EPI_ISL_9662999, EPI_ISL_9663000, EPI_ISL_9663001, EPI_ISL_9663002, EPI_ISL_9663003, EPI_ISL_9663004, EPI_ISL_9663005, EPI_ISL_9663006, EPI_ISL_9663007, EPI_ISL_9663008, EPI_ISL_9663009, EPI_ISL_9663010, EPI_ISL_9663011, EPI_ISL_9663012, EPI_ISL_9663013, EPI_ISL_9663014, EPI_ISL_9663015, EPI_ISL_9663016, EPI_ISL_9663017, EPI_ISL_9663018, EPI_ISL_9663019, EPI_ISL_9663020, EPI_ISL_9663021, EPI_ISL_9663022, EPI_ISL_9663023, EPI_ISL_9663024, EPI_ISL_9663025, EPI_ISL_9663026, EPI_ISL_9663027, EPI_ISL_9663028			
see above	State Institution "Kyiv City Center for Control and Prevention of Diseases of Ministry of Health of Ukraine"	L.V. Gromashevsky Institute of Epidemiology and Infectious Diseases of the NAMS of Ukraine	Alla M. Scherbinska; Anna Yakovleva; Eric Bortz; Ganna Kovalenko; Ian Goodfellow; Joel O. Wertheim; Luke Meredith; Mariia G. Liulchuk; Matthew Redlinger; Tetyana I. Vasylyeva; Viktoria I. Zadorozhna

We gratefully acknowledge the following Authors from the Originating laboratories responsible for obtaining the specimens, as well as the Submitting laboratories where the genome data were generated and shared via GISAID, on which this research is based.

All Submitters of data may be contacted directly via www.gisaid.org

[illegible]

Accession ID	Originating Laboratory	Submitting Laboratory	Authors
EPI_ISL_733077, EPI_ISL_733078, EPI_ISL_733151, EPI_ISL_733433, EPI_ISL_733452, EPI_ISL_1400527, EPI_ISL_1400557			
see above	HELIX LLC	WHO National Influenza Centre Russian Federation	Andrey Komissarov; Anna Ivanova; Artem Fadeev; Daria Danilenko; Dmitry Bazhenov; Dmitry Lioznov; Elena Nabieva; Georgii Bazykin; Ksenia Safina; Kseniya Komissarova
EPI_ISL_3932047, EPI_ISL_3996685, EPI_ISL_3996686, EPI_ISL_3996687, EPI_ISL_3996688	Molecular Diagnostic Laboratory of Federal Budget Institution of Science at the Central Research Institute of Epidemiology of the Federal Service on Customers' Rights Protection and Human Well-being Surveillance	Group of Genomics and Postgenomic Technologies of Central Research Institute of Epidemiology	Akimkin V.G.; Berlina Y.Y.; Buharina A.Y.; Kaptelova V.V.; Kondrasheva L.Y.; Korneenko E.V.; Nadtoka M.I.; Roev G.V.; Saenko S.S.; Samoilov A.E.; Shipulina O.Y.; Sinitsyn S.O.; Smirnova Y.S.; Speranskaya A.S.; Vyhodceva A.V.
EPI_ISL_2031957	Molecular diagnostic laboratory of Federal Budget Institution of Science "Central Research Institute of Epidemiology" of The Federal Service on Customers' Rights Protection and Human Well-being Surveillance	Group of Genomics and Postgenomic Technologies of Central Research Institute of Epidemilology	Akimkin V.G.; Kaptelova V.V.; Kondrasheva L.Y.; Korneenko E.V.; Nadtoka M.I.; Saenko S.S.; Samojlov A.E.; Shipulina O.Y.; Sinicyn S.O.; Smirnova Y.S.; Speranskaya A.S.; Tivanova E.V.
EPI_ISL_3101330, EPI_ISL_3101347, EPI_ISL_4536678, EPI_ISL_4536683, EPI_ISL_4536741, EPI_ISL_4536770, EPI_ISL_4558178, EPI_ISL_5306854, EPI_ISL_6642293, EPI_ISL_6642294, EPI_ISL_6642295, EPI_ISL_6642296, EPI_ISL_6642297, EPI_ISL_6901054, EPI_ISL_6901055, EPI_ISL_6901056, EPI_ISL_6901057, EPI_ISL_6901058, EPI_ISL_6901059, EPI_ISL_6901060, EPI_ISL_6901061, EPI_ISL_6901062, EPI_ISL_6901063, EPI_ISL_6901064, EPI_ISL_6901065, EPI_ISL_6901066, EPI_ISL_6901067, EPI_ISL_6901068, EPI_ISL_6901069, EPI_ISL_6901070, EPI_ISL_6901071, EPI_ISL_6901072, EPI_ISL_7688591, EPI_ISL_7688592, EPI_ISL_7688593, EPI_ISL_7688594, EPI_ISL_7688595, EPI_ISL_7688596, EPI_ISL_7688597, EPI_ISL_7688598, EPI_ISL_7688599, EPI_ISL_7688600, EPI_ISL_7688601, EPI_ISL_7688602, EPI_ISL_7688603, EPI_ISL_7688604, EPI_ISL_7717358, EPI_ISL_7717549, EPI_ISL_7717550, EPI_ISL_7717572, EPI_ISL_7717584, EPI_ISL_7717617, EPI_ISL_8189424, EPI_ISL_8540303, EPI_ISL_8540330, EPI_ISL_8540337, EPI_ISL_11359717, EPI_ISL_11359718, EPI_ISL_11359719			
see above	Molecular diagnostic laboratory of Federal Budget Institution of Science "Central Research Institute of Epidemiology" of The Federal Service on Customers' Rights Protection and Human Well-being Surveillance	Group of Genomics and Postgenomic Technologies of Central Research Institute of Epidemiology	Akimkin V.G.; Azarova V.S.; Berlina Y.Y.; Borzova S.I.; Buharina A.Y.; Bulanenko V.P.; Cherkashina A.S.; Chudinov I.K.; Dohoyan A.Y.; Dolotova S.M.; Golubeva A.G.; Goncharov S.E.; Gul'boj E.Y.; Kaptelova V.V.; Kochetova E.V.; Kondrasheva L.Y.; Korneenko E.V.; Markina N.V.; Nadtoka M.I.; Noskova O.M.; Roev G.V.; Saenko S.S.; Samoilov A.E.; Selezov S.Y.; Shipulina O.Y.; Sinitsyn S.O.; Smirnova Y.S.; Solovieva E.D.; Speranskaya A.S.; Svetlichnyj D.V.; Valdohina A.V.; Vyhodceva A.V.; Zotova M.I.; Zuev S.N.
EPI_ISL_3122988, EPI_ISL_3122989, EPI_ISL_3122990, EPI_ISL_3122991, EPI_ISL_3122992, EPI_ISL_3122993, EPI_ISL_3122994, EPI_ISL_3122995, EPI_ISL_3122996, EPI_ISL_3122997, EPI_ISL_3122998, EPI_ISL_3122999, EPI_ISL_3123000, EPI_ISL_3123001, EPI_ISL_3123002, EPI_ISL_3123003, EPI_ISL_3123004, EPI_ISL_3123005, EPI_ISL_3123006, EPI_ISL_3123007, EPI_ISL_3123008			
see above	Republican Children’s Clinical Infectious Diseases Hospital	WHO National Influenza Centre Russian Federation	Alexey Masharsky; Andrey Komissarov; Artem Fadeev; Daria Danilenko; Dmitry Lioznov; Elena Nabieva; Georgii Bazykin; Kirill Varchenko; Ksenia Safina; Kseniya Komissarova; Maria Baturova; Maria Pisareva; Mikhail Bakaev; Nikita Yolshin; Oula Mansour; Tamila Musaeva; Veronika Eder
EPI_ISL_8007819, EPI_ISL_8007821, EPI_ISL_8007822, EPI_ISL_8007823, EPI_ISL_8007824, EPI_ISL_8007825, EPI_ISL_8007827, EPI_ISL_8783298, EPI_ISL_8783299, EPI_ISL_8783300, EPI_ISL_8783301, EPI_ISL_8783302, EPI_ISL_8783303, EPI_ISL_8783304, EPI_ISL_8783305, EPI_ISL_8783306, EPI_ISL_8783307, EPI_ISL_8783308, EPI_ISL_8783309, EPI_ISL_8783310, EPI_ISL_8783311, EPI_ISL_8783312, EPI_ISL_8783313, EPI_ISL_8783314, EPI_ISL_8783315, EPI_ISL_8783316			
see above	Sevastopol Municipal Infectious Diseases Hospital	WHO National Influenza Centre Russian Federation	Andrey Komissarov; Artem Fadeev; Daria Danilenko; Dmitry Lioznov; Elena Nabieva; Georgii Bazykin; Kirill Varchenko; Ksenia Safina; Kseniya Komissarova; Maria Pisareva; Mikhail Bakaev; Nikita Yolshin; Oula Mansour; Tamila Musaeva; Veronika Eder
EPI_ISL_428901	State Research Center of Virology and Biotechnology VECTOR, Department of Collection of Microorganisms	State Research Center of Virology and Biotechnology VECTOR, Department of Collection of Microorganisms	Alexander N. Shvalov; Elena V. Gavrilova; Oleg V. Pyankov; Rinat A. Maksyutov; Sergey A. Bodnev; Tatyana V. Tregubchak
