# Supplementary material for: Tracking SARS-COV-2 variants using Nanopore sequencing in Ukraine in 2021
Source: Sci Rep. 2022 Sep 21;12:15749. doi: 10.1038/s41598-022-19414-y (PMC9491264; doi:10.1038/s41598-022-19414-y)
Supplement: Supplementary file 6 — Supplementary Information 6. [file 41598_2022_19414_MOESM6_ESM.docx]

**Supplementary text**

**Sequence quality**

Amplicon dropouts (absent regions regardless of coverage) were observed in most samples, leading to weak or missing regions (<20X read depth coverage). Notably, in sequences amplified with ARTIC nCoV-2019 v3 primers in August 2021 (N=24), amplicon 72 dropout, which covers the beginning of the Spike gene, was detected in all 21 Delta sequences, but not in the 3 Alpha sequences. The second major amplicon 64 (ORF1ab) dropout was detected for 12 Delta sequences.

Individual random amplicon dropouts were detected in samples with coverage <99% in the ORF1ab, S, M, ORF6, ORF8 and N genes. The information of amplicon number dropouts per each sequence are presented in Supplementary Table S1.

**Most common aa mutations**

All Alpha variant sequences (N=3) possess the same aa mutations in ORF1a: T1001I, A1708D, S3675-, G3676-, F3677-; in S: H69-, V70-, Y144-, N501Y, A570D, P681H, T716I, S982A, D1118H; in ORF8: Q27*, R52I, Y73C; and in N: D3L. The mutation I2230T in ORF1a was found only in two sequences. And only one sequence out of three has gained the common Alpha mutations such as R203K, G204R, S235F in N protein. For all 100 Delta variant sequences the following mutations were identified: P1000L in ORF1b; T19R, T478K, P681R, D950N in S protein; S26L in ORF3a; I82T in M; D63G in N.

The other common Delta mutations detected in S protein, such as E156-, F157-, and R158G, were present in 92 and L452R only in 97 out of 100 sequences. The R203M and D377Y mutations in N gene were gained in 95 and 99 Delta sequences, respectively. In ORF7a, the V82A mutation was present in 89 Delta sequences and T120I in 91. The most common co-occurred mutations P314L in ORF1b/RdRp and D614G in S protein for both Alpha and Delta VOC were present in 102 and 103 sequences, respectively (Figure 2 and Supplementary Table S1).

**PANGO lineages**

From the Oxford Nanopore sequences generated in this study, among the 100 Delta (B.1.617.2-like) variant sequences 10 different Delta sub-lineages were assigned using the PANGO Lineages tool (Pango v.4.0.6 PLEARN-v1.8): AY.4 (N=19) and AY.5 (N=1) UK sub-lineages; AY.25 (N=1) USA lineage; AY.33 (N=2) sub-lineage circulating mostly in North-western Europe; AY.36 (N=11) which is predominantly in Nigeria, UK, and USA; AY.43 (N=2), AY.98.1 (N=1), AY.122 (N=45) and AY.126 (N=2) – four common European sub-lineages; AY.46 (N=2) African lineage. Three out of 100 sequences belong to the Alpha (B.1.1.7-like) variant, and one of them was assigned as B.1.1.7-like+ E484K sub-variant.
